# Emerging and re-emerging zoonotic viral diseases in Southeast Asia: One Health challenge

**DOI:** 10.3389/fpubh.2023.1141483

**Published:** 2023-06-13

**Authors:** Paola Mariela Saba Villarroel, Nuttamonpat Gumpangseth, Thanaphon Songhong, Sakda Yainoy, Arnaud Monteil, Pornsawan Leaungwutiwong, Dorothée Missé, Sineewanlaya Wichit

**Affiliations:** ^1^Department of Clinical Microbiology and Applied Technology, Faculty of Medical Technology, Mahidol University, Nakhon Pathom, Thailand; ^2^Viral Vector Joint Unit and Joint Laboratory, Mahidol University, Nakhon Pathom, Thailand; ^3^Plateforme de Vectorologie, BioCampus, University of Montpellier, CNRS, INSERM, Montpellier, France; ^4^Department of Microbiology and Immunology, Faculty of Tropical Medicine, Mahidol University, Bangkok, Thailand; ^5^MIVEGEC, University of Montpellier, CNRS, IRD, Montpellier, France

**Keywords:** Southeast Asia, zoonoses, emerging and re-emerging viral diseases, drivers, One Health, epidemiology

## Abstract

The ongoing significant social, environmental, and economic changes in Southeast Asia (SEA) make the region highly vulnerable to the emergence and re-emergence of zoonotic viral diseases. In the last century, SEA has faced major viral outbreaks with great health and economic impact, including Severe Acute Respiratory Syndrome Coronavirus 2 (SARS-CoV-2), arboviruses, highly pathogenic avian influenza (H5N1), and Severe Acute Respiratory Syndrome (SARS-CoV); and so far, imported cases of Middle East Respiratory Syndrome Coronavirus (MERS-CoV). Given the recent challenging experiences in addressing emerging zoonotic diseases, it is necessary to redouble efforts to effectively implement the “One Health” initiative in the region, which aims to strengthen the human-animal–plant-environment interface to better prevent, detect and respond to health threats while promoting sustainable development. This review provides an overview of important emerging and re-emerging zoonotic viral diseases in SEA, with emphasis on the main drivers behind their emergency, the epidemiological situation from January 2000 to October 2022, and the importance of One Health to promote improved intervention strategies.

## 1. Introduction

Emerging and re-emerging infectious diseases are defined as recently recognized or evolved, or previously identified that have shown significant changes in their geographic, host or vector range (1). More than 60% of the emerging infectious diseases are zoonoses originating from domestic animals, poultry, livestock, and increasingly (71.8%) from wildlife species (2).

Zoonoses are caused by various pathogens, such as bacteria, viruses, parasites or prions that are naturally transmitted from vertebrate animals to humans during spillover events. In particular, viral infections pose a major threat to human health, as they can be transmitted by aerosol, direct contact with animals or their fluids, through food or vectors (3), and it is estimated that more than 1.6 million unknown viral species of mammalian and waterfowl can infect humans, of which up to half have zoonotic potential (4).

Zoonotic transmission involves the interaction of a pathogen and at least two host species: (a) a natural reservoir, infected with the pathogen and often asymptomatic (shedding the pathogen), (b) a recipient host, presenting the disease (infected with the pathogen from a different host), and (c) an intermediate host, that may or may not be present, acting as a bridge or mixing vessel (vertebrate or invertebrate vector). Pathogens can be transmitted to the recipient host (humans) directly from the natural reservoir, from the intermediate vertebrate or invertebrate host, or from the environment, resulting in transmission to humans without spread (“dead-end spillover”), or in adaptation for human-to-human transmission (5). Although these events are relatively rare, in the last century, outbreaks of emerging and re-emerging viral zoonoses have increased in frequency and magnitude with significant human and animal health impacts, as well as incalculable and far-reaching economic consequences, as a result of the intensification of the animal-human interface, driven primarily by anthropogenic factors (6).

Their unpredictable emergence, their potential to cause severe diseases in humans and animals, and the frequent absence of effective vaccines and antiviral treatments, make their containment difficult. Therefore, our ability to predict and prevent future outbreaks depends on recognizing, understanding, and mitigating this complex and multifactorial process, which involves the interaction of animals, environment, pathogens, and humans, creating a favorable environment for interspecies transmission. However, to effectively achieve these actions, collaboration and transdisciplinary partnerships are required.

**Figure 1:**
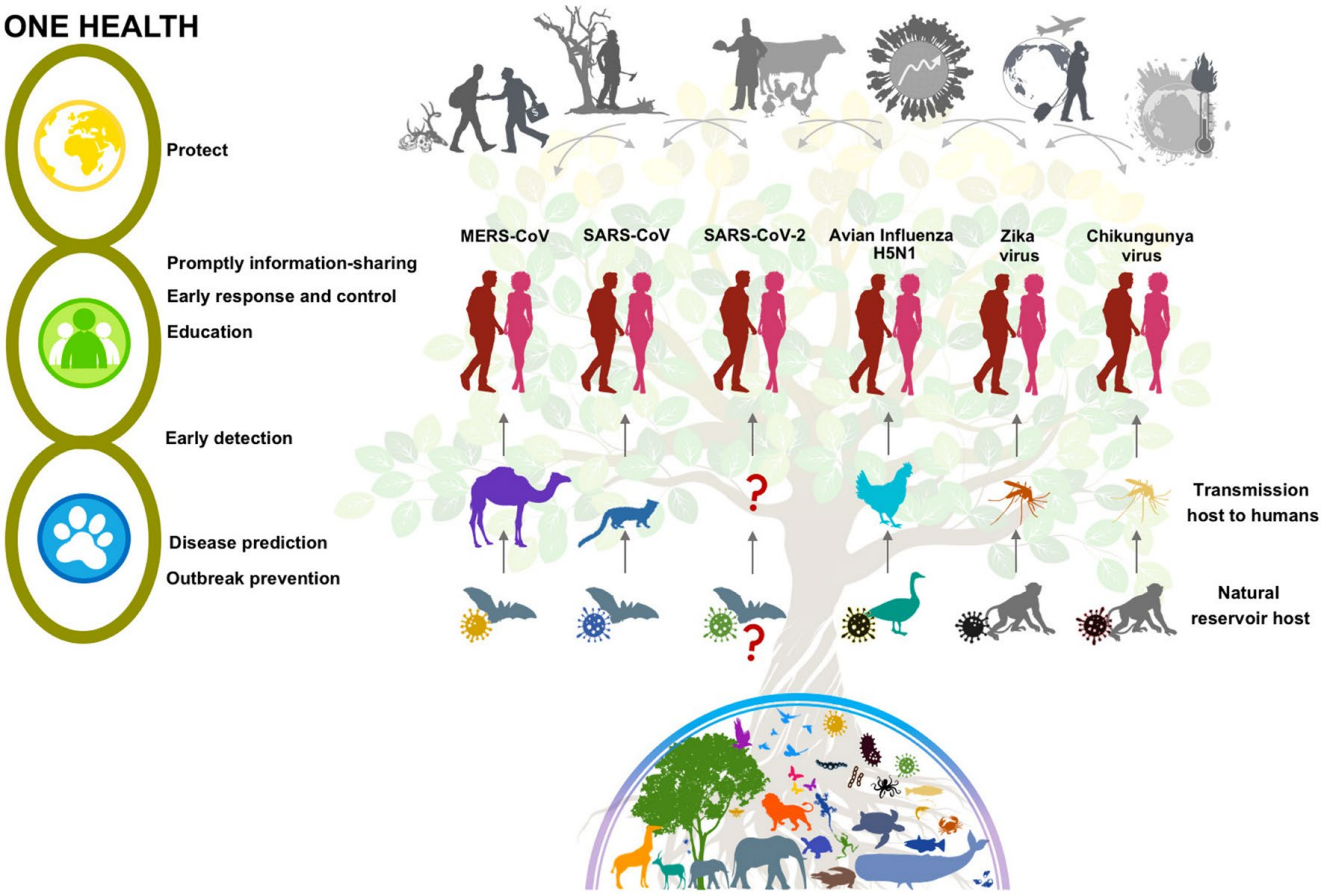
Emerging and re-emerging zoonotic viral diseases. Drivers, reservoir hosts, transmission to humans, and One Health action. Wildlife trade and consumption, deforestation, agriculture and meat production, population growth and urbanization, global travel, and climate change are well documented drivers that have contributed to the emergence and re-emergence of zoonotic viral diseases. Zoonotic spillover events are a complex mechanism that requires the interaction of a natural animal reservoir infected by a pathogen and often asymptomatic, a recipient host, which presents the disease, and a transmitting or intermediate host (vertebrate or invertebrate vector) that may or may not be present, acting as a bridge or serving as a mixing vessel. In the last century, several zoonotic viral diseases have emerged or re-emerged including: Middle East Respiratory Syndrome (MERS-CoV), Severe Acute Respiratory Syndrome (SARS-CoV) and Severe Acute Respiratory Syndrome Coronavirus 2 (SARS-CoV-2): Like SARS and MERS, SARS-CoV-2 has been hypothesized to have evolved from a strain found in bats. The main intermediate animal host responsible for human infection of SARS-CoV is the palm civet, for MERS-CoV the dromedary camel, and for SARS-CoV-2 it is still unknown. Highly pathogenic avian influenza (HPAI) H5N1: Wild waterfowl are the natural reservoir of low pathogenic avian influenza (LPAI) viruses, when transmitted to terrestrial poultry, LPAIVs can mutate into HPAI and be transmitted to humans. Zika virus (ZIKV): Transmission includes the sylvatic cycle, including non-human primates and arboreal canopy-dwelling *Aedes* mosquito species, and the urban cycle, including humans and mainly *A. aegypti* (the most competent) and *A. albopictus* mosquitoes. Chikungunya virus (CHIKV): Transmission includes the sylvatic cycle among forest-dwelling *Aedes* spp. mosquitoes and mainly non-human primates in Africa; and the urban cycle, maintained by mosquitoes (*A. aegypti* and *A. albopictus*) carrying the virus from human to human. One Health is a collaborative, multisectoral, and transdisciplinary approach that reinforces the human-animal–plant-environment interface to better prevent, predict, detect, and respond to health threats.

The World Health Organization (WHO), World Organization for Animal Health (OIE), and Food and Agriculture Organization (FAO), belonging to the Tripartite collaboration, have been working together for years, and in 2022 became quadripartite with the support of the United Nations Environment Program (UNEP) (7), to mainstream “One Health,” defined by WHO as “an approach to designing and implementing programs, policies, legislation and research in which multiple sectors communicate and work together to achieve better public health outcomes” (8). This approach supports countries to improve prevention, monitoring, detection, control and containment of zoonotic diseases while contributing to sustainable development (7) (Figure 1 illustrates the spillover events of selected zoonotic viral diseases from the natural reservoir to humans, influenced by drivers that promote their emergence and re-emergence, and the “One Health” initiative).

Southeast Asia (SEA) is a sub-region of Asia, within the tropical climatic zone, comprising the Association of Southeast Asian Nations (ASEAN) (Brunei, Singapore, Malaysia, Thailand, the Philippines, Indonesia, Vietnam, Lao People’s Democratic Republic (PDR), Cambodia, and Myanmar), and one observer state (Timor-Leste) (9). The region is politically, culturally, and socioeconomically diverse, undergoing major environmental, economic, and social changes (10), which have triggered a number of emerging and re-emerging zoonotic viral diseases during the last century (Table 1). The region is increasingly embracing “One Health.” However, there are still significant barriers that vary from country to country and hinder its successful implementation.

**Table 1:** Emerging and re-emerging zoonotic viruses in Southeast Asia in the XXI century (January 2000–October 2022).

Virus	Year of first human case* Year of major outbreak** (per country)	Country	Reference
SARS-CoV	2003	Vietnam	(11)
		Singapore
		Thailand
		The Philippines
		Indonesia
		Malaysia
Influenza A(H5N1)	2003	Vietnam	(12)
Influenza A(H5N1)	2004	Thailand	(12)
Influenza A(H5N1)	2005	Indonesia	(12)
		Cambodia
Dengue virus	2007	Cambodia	(13)
Influenza A(H5N1)	2007	Lao PDR	(12)
		Myanmar
Chikungunya virus	2008	Malaysia	(14)
Chikungunya virus	2009	Thailand	(15)
Ebola	2009	The Philippines	(16)
Influenza A(H1N1)	2009	All SEA countries	(16)
Chikungunya virus	2009	Myanmar	(17)
		Indonesia	(18)
Dengue virus	2013	Thailand	(15)
		Lao PDR	(19)
Chikungunya virus	2013	Singapore	(21)
		Lao PDR	(20)
Dengue virus	2014	Brunei	(22)
MERS-CoV	2014	Malaysia	(23)
MERS-CoV	2015	Thailand	(23)
		The Philippines
Dengue virus	2015	Myanmar	(24)
Dengue virus	2016	Indonesia	(25)
Zika virus	2016	Singapore	(26)
		Thailand	(15)
		Vietnam	(105)
Chikungunya virus	2017	The Philippines	(27)
Dengue virus	2019	Malaysia	(29)
		The Philippines	(28)
Monkeypox	2019	Singapore	(16)
SARS-CoV-2	2020	All SEA countries	(30)
Chikungunya virus	2020	Cambodia	(31)
Dengue virus	2020	Singapore	(32)
Monkeypox	2022	Vietnam	(33)
		Thailand
		The Philippines
		Indonesia
Dengue virus	2022	Vietnam	(32)
		Timor-Leste

SARS-CoV, Severe Acute Respiratory Syndrome; MERS-CoV, Middle East Respiratory Syndrome; SARS-CoV-2, Severe Acute Respiratory Syndrome Coronavirus 2; PDR, People’s Democratic Republic; SEA, Southeast Asia.

*Year of first human case: SARS-CoV, Influenza A(H5N1), Ebola, Influenza A(H1N1), MERS-CoV, Monkeypox, SARS-CoV-2.

**Year of major outbreak: Zika virus, dengue virus and Chikungunya virus.

The present review provides an update on emerging and re-emerging zoonotic virus diseases in SEA on (1) the drivers of their emergence, (2) the epidemiology of Severe Acute Respiratory Syndrome (SARS-CoV), Highly Pathogenic Avian Influenza (HPAI) H5N1, Middle East Respiratory Syndrome Coronavirus (MERS-CoV), Chikungunya virus (CHIKV), Zika virus (ZIKV), and Severe Acute Respiratory Syndrome Coronavirus-2 (SARS-CoV-2) between January 2000 and October 2022, (3) The success story of HPAI H5N1 in Thailand, and (4) lessons learned from previous diseases, and One Health.

## 2. Drivers of zoonotic viral diseases in Southeast Asia

SEA is a hotspot for zoonotic diseases caused by changes in the modern human population dynamics that disrupt the environment, such as population growth and travel, deforestation, agriculture and meat production, wildlife consumption and trade, as well as climate change (34).

### 2.1. Population growth and travel

The population of SEA has grown considerably, from 360 million in 1980 to more than 680 million inhabitants in 2022, representing 8.6% of the total world population. Social development has led to a decline in fertility, from 4.5 births/woman in 1980 to 2.1 in 2021, and to an improvement in mortality rates, with life expectancy raising from 60.4 years old (yo) in 1980 to 70.2 yo in 2021. However, gaps remain wide between countries (35).

The region has highly populated countries, such as Indonesia, the fourth most populous in the world, and densely populated countries such as Singapore, ranking third worldwide with 8,700 people per km^2^ (35). Urbanization is another notable population trend, driven by access to higher education, job opportunities, and health security. Currently, half of the SEA population lives in urban areas, and it is expected to exceed 70% by 2050 (36).

Migration has also emerged as a significant factor influencing population dynamics. Approximately 9.2 million migrants of working age live in Singapore (37% of the total population), Brunei (25.5%), Malaysia (15.0%, including undocumented migrants), and Thailand (5.2%, including undocumented migrants), with 77.2% of them coming from other ASEAN countries (37).

Population growth is closely related to emerging and re-emerging viral zoonotic diseases in many ways, such as high human density allows diseases to spread faster; the number of human births, immunologically naïve individuals, increases the risk of re-emerging diseases or depletion of immunity when vaccines are available; and old adult populations may increase viral transmission due to the lower capacity of the immune system to contain diseases. On the other hand, other human requirements also increase the risk, including housing (urbanization of untouched ecosystems), which comes along with overcrowding and low-quality dwellings, and increased migration of people (6, 38).

Tourism is a major source of income in SEA, which has grown from 63 million visitors in 2009 to 139 million in 2019 (before COVID-19 emergency), according to the United Nations World Tourism Organization (UNWTO) (39, 40), with almost 40 million visitors in Thailand (5^th^ in the world), 26 million in Malaysia, and 19 million in Singapore in 2019, generating 147.6 billion U.S. dollars in tourism receipts in 2019 (41, 42).

This good connectivity allows the spread of diseases to other continents in short periods, the introduction of new vectors into suitable environments, or new pathogens into vector populations, amplifying the risk of new outbreaks or pandemics (39, 40).

### 2.2. Deforestation

SEA has around 15% of the world’s tropical forests, and at least four of the 25 biodiversity landscapes (43). Unfortunately, deforestation is a pressing problem throughout the region, where at least 1.2% of its forests are lost annually (44) driven mainly by farming (73%), and logging (19%), with the fear that more than 40% of total forest area could disappear by 2,100 (43, 45). For example, a large loss in tree cover has been observed from 2001 to 2021, where Indonesia has lost 28.6 million hectares (Mha) (18% decrease in tree cover since 2000), Malaysia 8.67Mha (29%), Myanmar 4.3Mha (10%), Lao PDR 4.05Mha (21%), and Cambodia 2.60Mha (30%) (46).

The most important cause of deforestation in SEA is palm oil production, which has increased enormously, accounting for 90% of global production (45% of plantations were forests in 1989). In particular, Indonesia is the main producer (followed by Malaysia), contributing to the country’s economy and providing job opportunities to 4 million people (45, 47, 48); consequently, it is the worst-affected country in the region, in addition to having experienced a massive fire in 2016 that accounted for 30% of all tree cover loss for that year and was reportedly deliberately started by small-scale farmers to clear lands. Cambodia, on the other hand, recently records the highest percentage of total forest loss worldwide due to poor forest management (between 2001 and 2010 88,000 ha/year, and 2011–2021: 155,000 ha/year) (46, 49, 50).

Among the many consequences of deforestation and forest degradation, including loss of biodiversity [SEA among the most threatened regions, at least 221 terrestrial and freshwater vertebrates are critically endangered (51)], climate change [12–20% of global greenhouse gas emissions (GHG)], and loss of soil fertility, are zoonotic diseases, which particularly affect countries in the intertropical zone with high forest cover. It causes environmental stress on wildlife, an impact on the reservoir host (e.g., survival of generalist and opportunistic species along with their pathogens) and/or vector populations dynamics that favor transmission, as well as increased animal-human interaction (52–54).

### 2.3. Agriculture expansion, meat production, wildlife consumption and trade

Agriculture in SEA is an important source of economy and livelihood, except for Singapore and Brunei, contributing to more than 10% of the gross domestic product (GDP), and employs one-third of all workers (55). The region is among the main producers of rice, vegetable oil, and sugar (56). Rice production is the main crop and accounts for 26% of global production and 40% of world exports, with Cambodia, Indonesia, Myanmar, the Philippines, Thailand and Vietnam being the main producers per crop (57). Maize is the second most produced cereal, which is also the primary source of feed for the poultry and livestock industry, and has the largest harvested area in Indonesia, the Philippines, Thailand, and Vietnam (58). Furthermore, Thailand has become the world’s leading exporter of rubber (56, 59).

Meat production has increased enormously; in particular, between 2009 and 2018 poultry farming has expanded by 56%, and pig farming has increased by 23%, especially in Vietnam and Thailand. Moreover, Indonesia is the largest egg producer (56, 59). At the same time, wild animals are hunted indiscriminately and removed from their natural habitat through legal and illegal trade. They are traded for collectibles, food (for some it represents status and prestige, believed to be “healthier” or simply for their “wild taste”) and served in restaurants in Vietnam and Cambodia, but also as sale items, pets, medicinal, in open-air wet markets, or through online platforms and social media (53, 60). To illustrate, more than 3,000 parts and products of the critically endangered Helmeted Hornbill were seized, especially in Indonesia between 2010 and 2019, over 96,000 kg of pangolin scales in Malaysia, Singapore and Vietnam between 2017 and 2019, and 45,000 live birds in Indonesia in 2018–2019 (51).

Agriculture is responsible for GHG emissions, loss of biodiversity, deforestation, increased water demand, increased release of reactive nitrogen into the environment, and allows pathogens to jump species by enabling the movement of animals, the exchange of products and services, the confinement of animals in close contact and stressful conditions, and through the consumption of wild meat (61).

### 2.4. Climate change

The region is one of the most vulnerable to climate change, facing warming trends and a possible alteration of the South Asian monsoon pattern. These changes are primarily attributed to the use of fossil fuels, deforestation, and agricultural practices (62). Annual temperatures have increased by about 0.6°C per decade over the past 100 years (63), and weather events have increased in number and intensity. According to the 2022 world risk index report, the Philippines (1st worldwide), Indonesia (3rd), Myanmar (6th), and Vietnam (12th) had the highest estimated disaster risk (64), while according to the long-term climate risk index between 2000 and 2019, Myanmar was the most affected country in SEA and the second in the world, where Cyclone Nargis in 2008 was the worst natural disaster ever recorded in the country, and at least the second deadliest globally, responsible for 140,000 deaths and catastrophic destructions, followed by the Philippines which is recurrently affected by tropical cyclones such as Pablo (Bopha) in 2012, Yolanda (Haiyan) in 2013, Ompong (Mangkhut) in 2018, and Odette (Rai) in 2021 (65).

The increase in global temperature or the length of the seasons also affect the geographic distribution and density of species. Particularly, it influences the transmission dynamics of vector-borne infections, increasing the survival, reproduction, and abundance of vector populations (6).

## 3. Epidemiology of emerging and re-emerging viral zoonoses in Southeast Asia (January 2000–October 2022)

### 3.1. Severe acute respiratory syndrome coronavirus

In November 2002, SARS-CoV first emerged in Guangdong Province, China. Bats have been recognized as the natural reservoir, and the palm civet, as the intermediate animal host (Figure 1). Early cases were detected in patients who lived near a market or were food handlers (66, 67). The epidemic spread within Guangdong with a high rate of transmission among health care workers (HCWs), before spreading to Hong Kong in February 2003, through an infected HCW who stayed in a hotel and caused infection in at least 16 guests and visitors. The movement of infected people, caused other outbreaks within and outside the country (68). The SARS pandemic ended in July 2003, and caused over 8,000 infections and 774 deaths in 29 countries, with a case fatality rate (CFR) of 9.5%, and about 50.0% among patients aged >65 years. Five additional zoonotic cases were confirmed between December 2003 and January 2004 (11, 69).

**Figure 2:**
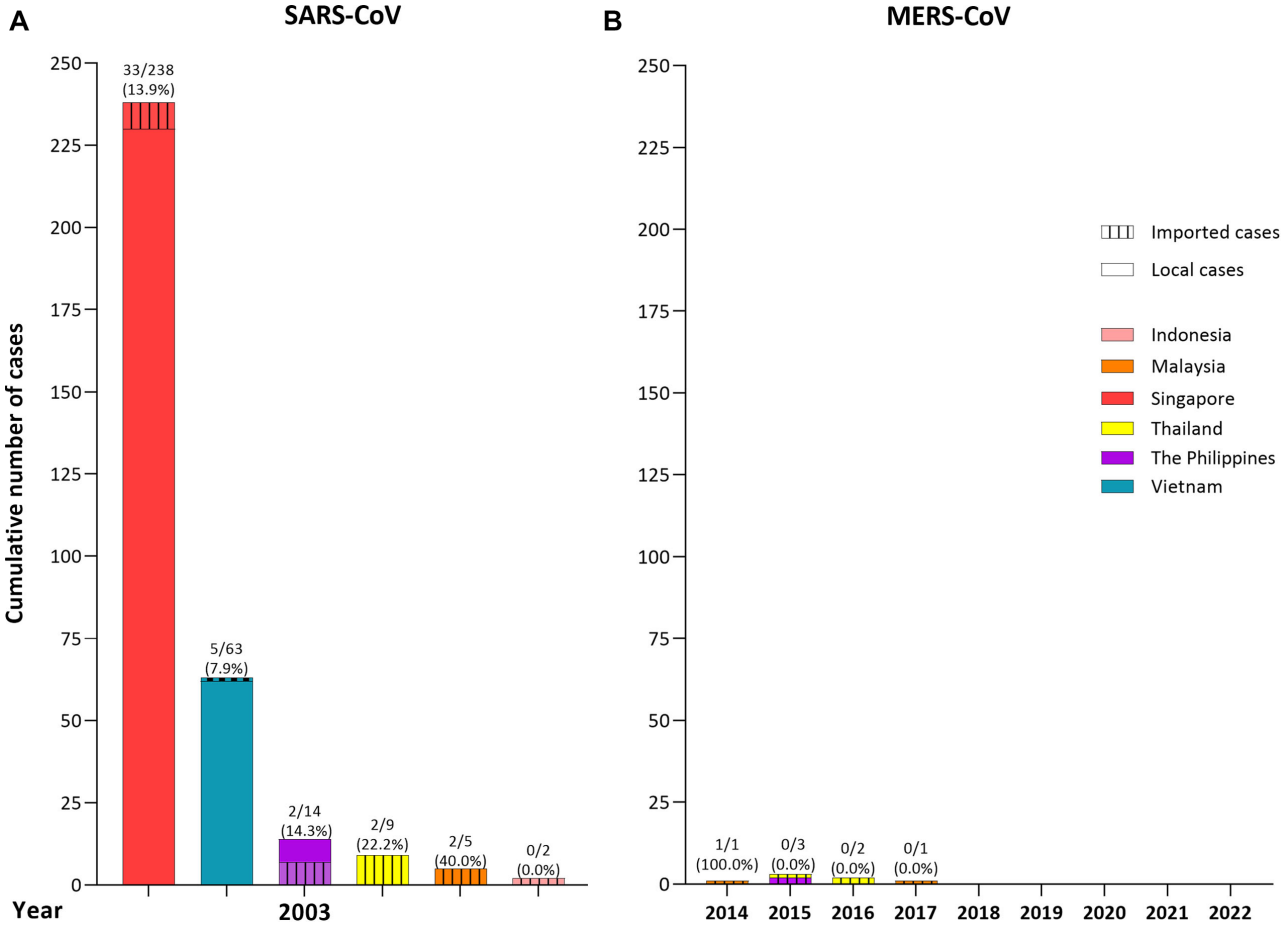
Cumulative number of cases (local or imported) of human Severe Acute Respiratory Syndrome (SARS), and Middle East Respiratory Syndrome Coronavirus (MERS) in Southeast Asia (SEA) per country and per year (until October 2022). **(A)** SARS: 331 human cases (4.1% of global cases) and 44 deaths were reported between February and May 2003 in 6 countries. Imported cases and local transmission were reported in Singapore, Vietnam, and the Philippines, while only imported cases were reported in Thailand, Malaysia, and Indonesia. **(B)** MERS: 7 imported cases (0.3% of global cases) with one death were reported between 2014 to 2017 in 3 countries, Thailand, Malaysia, and the Philippines. The number in bar charts represent: number of deaths/total number of cases per country (% of mortality).

Six countries in SEA reported a total of 331 human cases of SARS-CoV infection (4.1% of global cases) and 44 deaths (CFR = 13.3%) between late February and May 2003. Transmission was initiated by the hotel guests and caused mainly nosocomial infections in Singapore (total cases *n* = 238; imported = 8; CFR = 13.9%; HCWs = 41.0%) (68), in Vietnam (total *n* = 63; imported = 1; CFR = 7.9%; HCWs = 57.0%) (11, 68), and in the Philippines (total *n* = 14; imported = 7; CFR = 14.3%; HCWs = 28.6%), the latter caused by a nursing assistant, derived from an outbreak in Toronto, Canada (70). However, Thailand (*n* = 9, CFR = 22.2%), Malaysia (*n* = 5; CFR = 40.0%), and Indonesia (*n* = 2; CFR = 0.0%) reported only imported cases (11) (Figure 2A).

### 3.2. Middle East respiratory syndrome coronavirus

In June 2012, a patient with MERS was first reported in Jeddah, Saudi Arabia. However, a cluster of undiagnosed severe respiratory illness among HCWs in Jordan in April 2012 was later confirmed to be caused by MERS. Like SARS, MERS evolved from bats (71), and the dromedary camel acted as the intermediate animal host responsible for human infection (Figure 1) (72). The disease has spread within (e.g., Qatar) and outside the Middle East, including the Republic of Korea, where in 2015 a traveler caused a large outbreak in 16 healthcare settings (73, 74). As of 31 October 2022, 2,600 human cases of MERS have been reported (Saudi Arabia = 84.3% of cases), with 935 deaths (CFR = 36.0%) in 27 countries. Transmission occurred mainly among patients in healthcare settings (62–79%) (73, 75).

Between 2014 and 2017, seven imported human cases (0.3% of global cases) with one death (CFR = 14.3%) of MERS were confirmed in three SEA countries. Three cases in Thailand, the first and second were in older men from Oman in June 2015 and June 2016, who arrived for other medical reasons, and the third in July 2016 in a young Kuwaiti man who arrived for vacation (76), two cases in Malaysia, in men returning from a pilgrimage in Saudi Arabia, in April 2014 (the first death), and in December 2017 (77, 78), and two in the Philippines, in January 2015, in a Filipino nurse working in Saudi Arabia (79), and in July 2015 in a male from Finland who traveled to Saudi Arabia and United Arab Emirates before arriving in the country (80) (Figure 2B).

### 3.3. Chikungunya virus

Chikungunya virus (CHIKV) was first isolated in Tanzania in 1952 from the serum of a febrile patient. Since then and for the next 50 years, sporadic cases were reported in Africa and Asia. CHIKV has recently been transmitted globally on all continents except Antarctica, affecting millions of people each year, especially in all tropical and several subtropical areas (81, 82). Transmission includes both sylvatic and urban cycles. The sylvatic cycle among forest-dwelling *Aedes* spp. mosquitoes and mainly non-human primates in Africa (including Guinea baboons, Chacma baboons, African green monkeys, patas monkeys, red-tail monkeys, guenons, bushbabies, and mandrills (83)), and the urban cycle, maintained by mosquitoes (*Aedes aegypti* and *Aedes albopictus*) carrying the virus from human to human, observed in the Americas, Asia, the Indian Ocean, and Europe (84, 85) (Figure 1).

**Figure 3:**
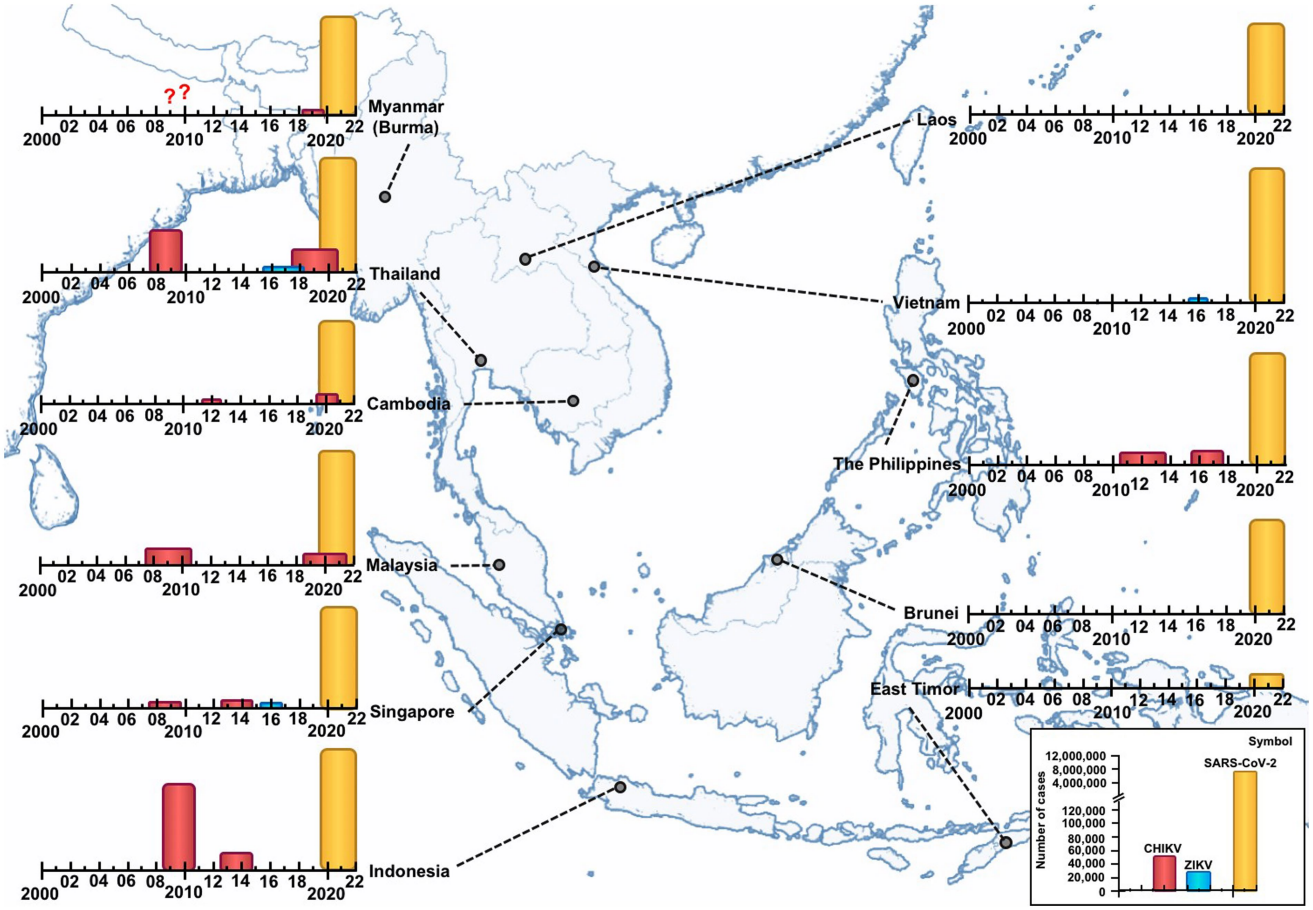
Epidemics of Chikungunya virus (CHIKV) (two major), Zika virus (ZIKV), and Severe Acute Respiratory Syndrome Coronavirus 2 (SARS-CoV-2) in Southeast Asia (SEA) per country (January 2000–October 2022). CHIKV has caused several outbreaks of varying magnitude and scope. The cumulative number of cases of the two major recorded outbreaks per country is as follows: Indonesia: 2009–2010 = 135,000 cases and 2013–2014 = ~22,500 cases. Thailand: 2008–2009 = 54,000 cases and 2018–2020 = 17,000 cases. Malaysia: 2008–2010 = 10,500 cases and 2019–2021 = ~5,000 cases. The Philippines: 2011–2013 = 2,800 cases and 2016–2018 = 11,500 cases. Cambodia: 2011 = 190 cases and 2020= > 6,000 suspected cases. Singapore: 2008–2009 = 1,059 and 2013–2014 = 1,241. Myanmar and Lao PDR = few cases. Vietnam, Brunei and East-Timor: no data. ZIKV outbreaks were limited and were reported between 2016 and 2018, in Thailand (2,300 cases), Singapore (458 cases), and Vietnam (265 cases). SARS-CoV-2: East-Timor, Cambodia and Lao People’s Democratic Republic reported the lowest number of confirmed cases (23,305, 137,995 and 150,000 cases, respectively); in contrast, Indonesia and Vietnam reported the highest number, with 7 and 11 million, respectively. ??: no data of cases.

In SEA, CHIKV emerged in Bangkok, Thailand in 1958 (although evidence suggests earlier transmission in Indonesia (86)), followed by other minor outbreaks or sporadic cases in other countries such as Cambodia (1961), the Philippines (1965), Vietnam (1966–1967), and Indonesia (official report 1972), with no major outbreaks between the 1980s and 2000s (81, 87). CHIKV re-emerged in the region in the 21st century. Some countries suffered large and multiple outbreaks, mainly after 2008, while others experienced low-level circulation. Indonesia has been the most affected country (25), followed by Thailand (15), Malaysia (14, 88), the Philippines (88–90), Cambodia (31, 91), and Singapore (92). While Myanmar (17, 93), and Lao PDR (20, 94) reported minor outbreaks or sporadic cases, and no available data are available for Vietnam, Brunei and Timor-Leste (the number of cases of the two largest outbreaks per country is reported in Figure 3). However, the true burden of CHIKV virus disease remains unknown. The number of cases is often underreported, due to the limited laboratory diagnosis, lack of accurate reports to health authorities, limited surveillance programs, and co-circulation with dengue virus on some occasions that may mask CHIKV infections (20, 95).

### 3.4. Zika virus

Zika virus (ZIKV) was isolated in 1947 from a sentinel rhesus monkey in the Zika forest of Uganda, and in humans in 1952 in Nigeria. The virus is maintained in two cycles: the sylvatic cycle, including non-human primates and arboreal canopy-dwelling *Aedes* mosquito species, and the urban cycle, including humans and mainly *A. aegypti* (the most competent) and *A. albopictus* mosquitoes (96) (Figure 1).

Serological evidence suggests that ZIKV circulated a low but at sustained levels in African countries from 1945 to 2014, and in Asian territories from 1952 to 1997 (97, 98). The first known outbreak occurred in 2007 in the Yap State of Micronesia, followed by the Pacific Islands in 2013–2014. Subsequently, major outbreaks occurred in Latin America and the Caribbean between 2015 and 2016. ZIKV has affected more than 87 countries and territories worldwide (99, 100).

ZIKV has been circulating in SEA since at least the 1950s based on neutralization assays. However the first human case was confirmed in 2010 in Cambodia (101). Epidemiological data are limited, with outbreaks reported between 2016 and 2018 in Thailand (2,300 cases) (102, 103), Singapore (458 cases) (26), and Vietnam (265 cases) (104, 105). Studies evidence low-level circulation in the Philippines, Myanmar, Lao PDR, Cambodia, Malaysia, and Indonesia (101, 102, 106–108), and no data are available for Brunei and Timor-Leste (Figure 3).

Overall, laboratory-confirmed and probable ZIKV cases do not estimate the total number of cases, which are often underestimated due to asymptomatic and pauci-symptomatic cases and combined with similar clinical presentation of other diseases such as dengue fever. In addition to misinterpretation of serological data as consequence of extensive cross-reactivity between flaviviruses that must be confirmed by seroneutralization tests (101).

### 3.5. Severe acute respiratory syndrome coronavirus 2

Recently, in December 2019, SARS-CoV-2 the etiologic agent of COVID-19 (Coronavirus disease 2019) emerged in Wuhan City, Hubei Province, China, with unidentified pneumonia cases associated with a wholesale seafood market. The virus is believed to have evolved from bats (71). However, for a bat viral pathogen to successfully emerge in humans, it usually requires an intermediate host, which remains unknown in the case of SARS-CoV-2 (Figure 1) (72). The virus spread rapidly to all continents, leading the WHO to declare a global pandemic in early March 2020 (67). To date, the pandemic has caused more than 629 million confirmed cases and 6.5 million deaths worldwide (109).

SEA was one of the first affected regions, with cases reported in January 2020 in Thailand, Vietnam, Malaysia, Cambodia, Singapore, and the Philippines (110). To date, more than 35 million confirmed cases (Figure 3) have been reported with a CFR of 0.7. Currently, Myanmar has the highest CFR (3.1%), while Brunei and Singapore have the lowest CFR (~0.1%) (30). Among cumulative cases per 100,000 inhabitants, Brunei and Singapore top the list. In contrast, Cambodia and Myanmar are at the bottom of the list (111). It should be noted that the number of cases is underestimated in several countries, due to underreporting of cases, related to poor testing, asymptomatic infections or mild symptoms (112).

Since December 2020, vaccines have played an important role in the COVID-19 pandemic. At this time, Brunei (99.9%) and Singapore (93.9%) have the highest number of fully vaccinated people, unlike Myanmar (51.2%) and Indonesia (62.4%), which have the lowest vaccination coverage (113).

### 3.6. Highly pathogenic avian influenza

H5N1 virus emerged in 1996 in farmed geese in Guangdong Province of southern China (People’s Rep. of), as the goose/Guangdong lineage (Gs/Gd), followed by an outbreak in poultry in Hong Kong in 1997 that resulted in the culling of 1.3 million chickens. The virus evolved from low pathogenic avian influenza (LPAI) viruses present in wild waterfowl (natural reservoir). LPAIVs, when transmitted to terrestrial poultry, can mutate into highly pathogenic avian influenza (HPAI), and can “spillback” to wild birds, which can carry and shed the viruses (114) (Figure 1). Since its emergence, the H5N1 subtype has evolved and diversified into multiple phylogenetic lineages (clades), given the segmented nature of the influenza viral genome that confers evolutionary advantages (115, 116).

In 2003, H5N1 reemerged and caused several outbreaks in SEA, including Vietnam, Thailand, Cambodia, Laos, Indonesia, Malaysia, and Myanmar, mainly among backyard poultry (61), and other Asian countries such as South Korea, Japan, Hong Kong, and China (117). Outside of Asia, the virus has spread to the Middle East, Africa, Europe, North America (Canada and United States of America), and recently to Latin America (118). Studies suggest that poultry and migratory birds were involved in the introduction into Asia and Africa, and migratory flyways of wild birds to Europe and America (119, 120).

Human cases were first reported in Hong Kong in 1997. The infection of three individuals returning from Fujian, China and the death of two of them marked the re-emergence in early 2003 (121). To date, it has caused 868 human cases (Egypt, 41.5% of cases) and 456 deaths (>50% mortality, varying between countries) in 21 countries worldwide, affecting mainly children and younger adults (122, 123).

**Figure 4:**
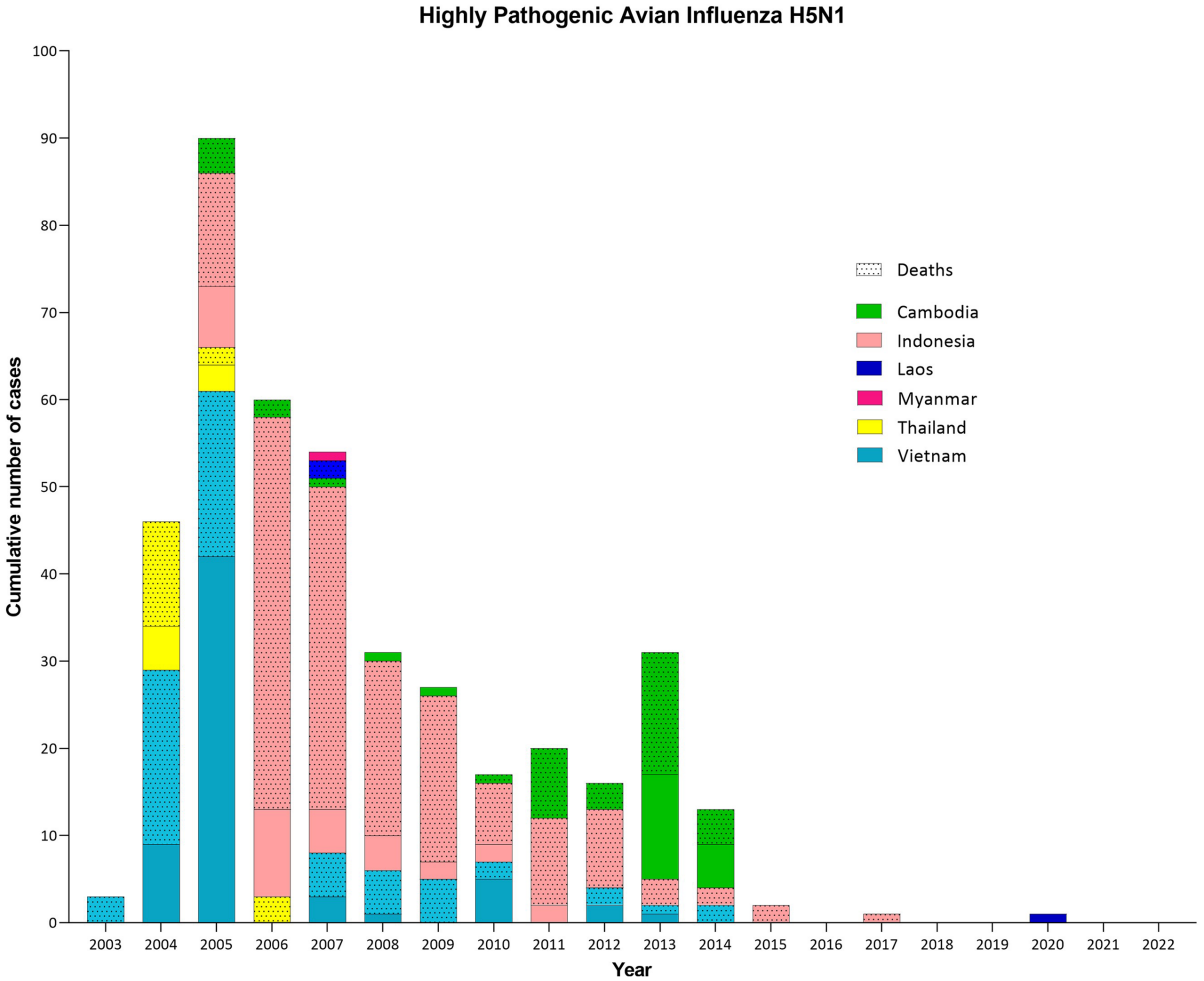
Cumulative number of confirmed human cases and deaths of Highly Pathogenic Avian Influenza H5N1 in Southeast Asia (SEA) per country and per year (until October 2022). Human cases of H5N1 in SEA represent almost half of the total number worldwide (412/868: 47.5%), with a high case fatality rate (69.9%). Between 2003 and 2020, cases were reported in 6/11 SEA countries, with 2005 being the most devastating year. Indonesia (200 cases and 168 deaths, CFR = 84.0%), Vietnam (127 cases and 64 deaths, CFR = 50.4%) and Cambodia (56 cases and 37 deaths, CFR = 66.1%) have been the most affected countries, reporting intermittent cases until at least 2014, unlike Thailand (25 cases and 17 deaths, CFR = 68.0%), which succeeded to report no human cases after 2006. In addition, Myanmar (1 case) and Lao PDR (3 cases and 2 deaths, CFR = 66.7%) confirmed some cases.

The epidemiological data of human H5N1 demonstrate the high percentage of cases described in SEA (*N* = 412), corresponding to 47.5% of global cases, and high mortality rates with almost 70.0%. Several countries were affected such Indonesia (200 cases), Vietnam (127 cases), Cambodia (56 cases), Thailand (25 cases), Lao PDR (3 cases), and Myanmar (1 case), mainly between 2004 and 2014 (12) (Figure 4). Most human cases had a history of close contact with poultry (e.g., backyard farming systems, live bird markets, consumption of diseased poultry). Although clusters of limited human-to-human transmission have occurred after prolonged exposure to a symptomatic infected person, sustained human-to-human transmission has not yet occurred (61).

## 4. The success story of highly pathogenic avian influenza in Thailand

Since 2003, the HPAI H5N1 virus has spread across SEA, causing unprecedented epidemics affecting poultry farmers, livelihoods, commercial poultry, tourism, and human health. In particular, Thailand has experienced several large epidemics during 2004 and 2005 in 60 of 76 provinces (124). However, the outbreaks were reduced to very low levels and no human cases were reported after 2006 due to effective control strategies (125).

### 4.1. Outbreak investigation

The virus was first confirmed on January 23, 2004 in Thailand, on a chicken farm in Suphanburi Province. This first wave lasted until May 2004, with 193 outbreaks in 42 of 76 provinces, affecting mainly chickens, but also broilers, layers, native chickens, ducks, geese, turkeys, ostriches, quail, and peacocks (126). The second large wave started on July 3, 2004, when H5N1 was confirmed on layer farms in Ayutthaya and Pathum Thani Provinces, and finished in March 2005 with a higher number of confirmed outbreaks (~1,492 outbreaks in 52 provinces), particularly in ducks and backyards chickens with poor hygiene. This higher number can be explained by the higher number of tests performed compared to the first wave (117). More than 62 million birds were either killed or culled for disease control (126). However, from July 2006 to 2011, there were few and limited outbreaks, mainly on small-scale backyard farms (124, 127).

**Figure 5:**
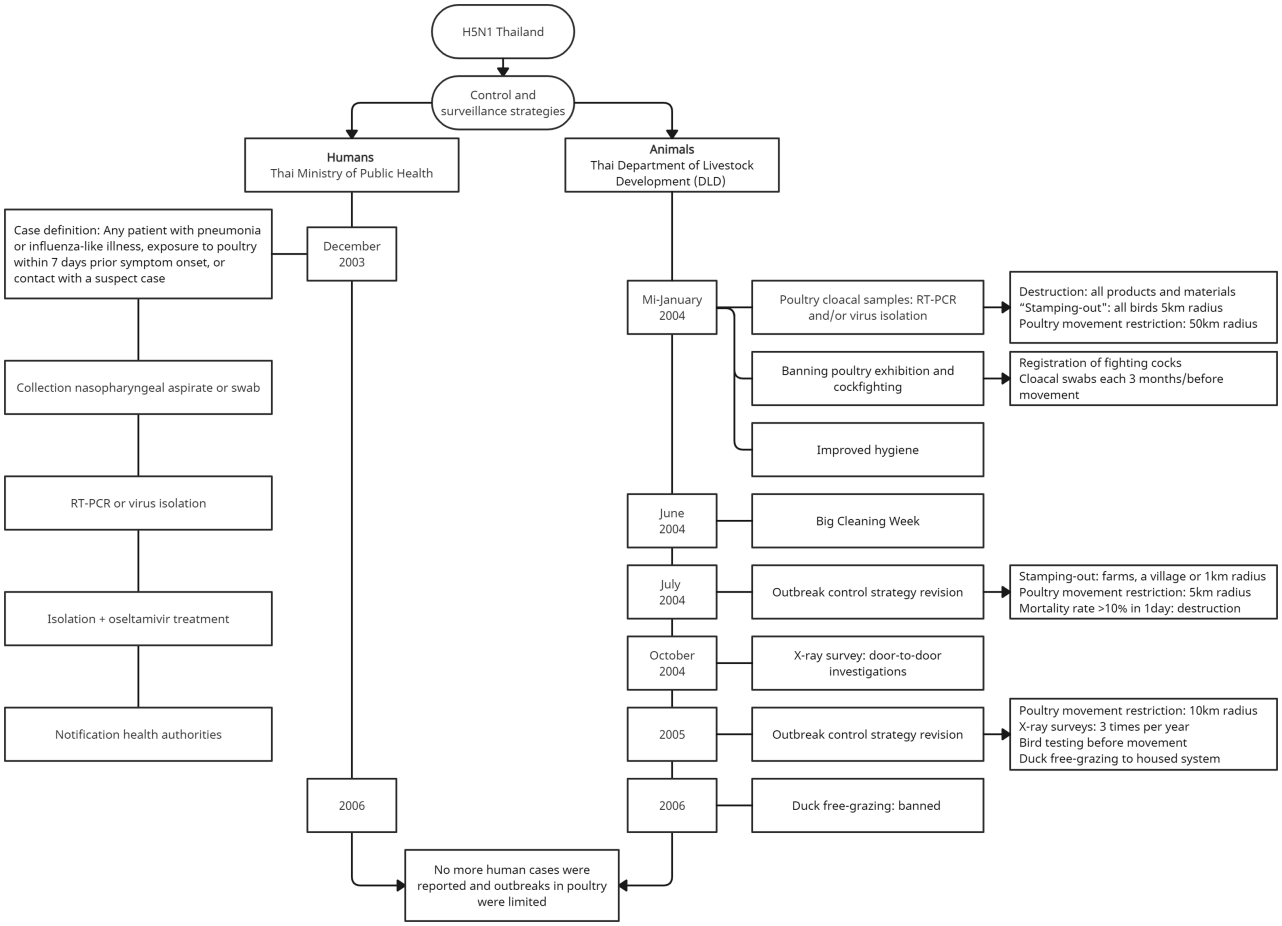
Highly pathogenic avian influenza H5N1 control and surveillance strategies in Thailand. The control and surveillance strategies implemented in Thailand during the large outbreaks in 2004–2005, limited the number of poultry outbreaks and human cases after 2006.

In response, a program throughout Thailand was launched in mid-January 2004 to detect cases in sick or dead poultry (117). The Thai Department of Livestock Development (DLD) implemented several strategies to control epidemics including disease investigation, animal movement control, pre-emptive culling (“stamping-out”), disinfection, surveillance and quarantine (117, 126, 128), as described in Figure 5. The Thai government compensated farmers for their losses, which encouraged farmers to report outbreaks. In addition, improved detection, better hygiene, and better response after the first wave contributed to the decrease in the number of outbreaks in poultry (129, 130). Surveillance results during the first week identified that the virus was already circulating in many types of poultry throughout the north and the south (130), stating that the delay in the identification was too long and contributed to the large scale of the outbreaks.

Thailand prohibited vaccination, although black-market vaccines may have been used (131). Unlike other countries like Vietnam that used inactivated vaccines to stop outbreaks, but after a year without outbreaks, the country faced a major outbreak in 2007 and sporadic outbreaks since then. This situation confirms the difficulties in maintaining good flock immunity in poultry populations (132). Studies have shown that the H5 subtype is less immunogenic (133). However, vaccination can be effective for outbreak control when accompanied by strict monitoring and testing (117).

The Ministry of Public Health confirmed the first human cases in children with severe progressive pneumonia from Suphanburi and Kanchanaburi Provinces on the same day of detection in poultry, through the national surveillance program to investigate human cases (134, 135) (Figure 5). Human outbreaks occurred mainly in Central Thailand, affecting children, between January–March and August–October 2004. In September 2004, a probable person-to-person transmission was identified in a family cluster (135).

### 4.2. Tracing the origin of avian influenza H5N1

Virus introduction into Thailand could have been *via* migratory birds (119); its expansion through the transport of poultry and poultry products, and the trade in wild birds; and its persistence caused by free-grazing ducks and rice cultivation (130). In Thailand, the high density of poultry populations (commercial and backyard poultry sectors with low biosecurity), live bird markets, bird migration from central and northern Asia to Thailand, and festivals related to poultry production and movement, played an important role in the spread of the initial large-scale outbreaks, and made disease control difficult in 2004 (117, 128, 136).

### 4.3. Breaking the transmission chain by using a One Health program

Thailand has recognized the need to establish a surveillance system since 2005, implementing strategies, such as the National Strategic Plan for Avian Influenza Control and Pandemic Influenza Preparedness in Thailand 2005–2007, launched by the Thai Government. This plan was then succeeded by the Second National Strategic Plan for Prevention and Control of Avian Influenza and Preparedness for Influenza Pandemic BE 2551–2,553 (AD 2008–2010), and finally, Thailand adopted “One Health” for emerging infectious diseases into the national strategic plan (2013–2016) (137–139).

Thailand innovates with a specific government unit, the Coordinating Unit for One Health (CUOH), which is the coordinating center for One Health activities. Several collaborators, such as government agencies, academic institutions, and the private sector, work together to maintain communication and promote activities (e.g., surveillance, control, conferences, and training) (137–139). For instance, in 2016, Thailand piloted a One Health avian influenza surveillance system, which demonstrated strengths but also encountered challenges (140).

## 5. Lessons from previous emerging and re-emerging viral zoonoses, and One Health

Since the early 2000s, zoonotic diseases have caused considerable economic and human impact, and have highlighted the importance of surveillance, prevention and control, as well as the importance of coordination between the human and the animal health sectors. However, the COVID-19 pandemic has exposed long-existing gaps in addressing emerging diseases, including: (a) preparedness and prompt response, (b) public health infrastructure, (c) effective and rapid risk communication, (d) research, and (e) political commitment, and national and international collaborations (141), and recognized the interdependence of humans, animals, plants and environment that reinforced the relevance of One Health (142).

One Health is a solution for sustainable and equitable future by protecting and restoring ecosystems, preserving human and animal health, and providing long-term economic benefits. This initiative requires mobilization, communication, coordination and collaboration across multiple sectors, disciplines, communities, and all levels of government (143).

SEA is committed to One Health, but despite the substantial progress made in recent years, it remains challenging to address illegal wildlife trade, corruption, insufficient political regulations, funding, population growth (e.g., culture and education), illegal logging, climate change, the preservation of forests, ecosystems and species, deforestation (economic pressure generated by the agricultural sector that limits policies), infrastructure and data sharing (in the most vulnerable regions) (143).

To better implement this approach, it is necessary to establish or strengthen cross-sectional and transdisciplinary working groups to share knowledge, challenges, needs and solutions, and thus develop effective actions and plans. Learn about existing One Health tools, resources and frameworks to improve public awareness, including rural and indigenous communities (e.g., create incentives). Identify One Health funding opportunities to receive support for sustainable projects. Promote information sharing and training among SEA countries, including communication, leadership and health diplomacy. Improve existing agreements by encouraging investment in sustainable strategies (143, 144).

Therefore, preventing the next zoonotic pandemic requires a critical shift toward a sustainable, cost-effective and integrated approach.

## 6. Concluding remarks

Zoonotic diseases do not respect national borders and can rapidly spread across regions and countries. Southeast Asia is a hotspot for the emergence and re-emergence of zoonotic viral diseases induced mainly by land use changes resulting from agriculture and population growth, which threaten biodiversity, forests, and climate. Over the last century, the region has experienced zoonotic viral outbreaks of varying magnitude and scope; in particular, apart from the SARS-CoV-2 pandemic, large epidemics of Chikungunya virus, highly pathogenic avian influenza H5N1, and SARS have been reported, causing serious concern and enormous health and economic impact.

To reduce the spread of viral zoonotic diseases in SEA, increasing multidisciplinary networks between countries is important. However, several factors including different cultures and traditions, surveillance and control systems disparities between SEA countries can limit the sharing of information and resources. Therefore, it is important to address these challenges and implement effective prevention measures tailored to the region’s specific needs and circumstances. The level of “One Health” education programs should increase, it may also involve educating the population about the causes and transmission of diseases and promoting healthy behaviors and practices that can help to prevent the spread of viral zoonotic diseases.

## Author contributions

PMSV, NG, and SW contributed to the conception, design of the study, and wrote the first draft of the manuscript. PMSV, NG, TS, SY, AM, PL, DM, and SW reviewed and edited the manuscript. All authors contributed to the manuscript revision, read, and approved the submitted version.

## Funding

This work (Grant No. RGNS 64-172) was supported by Office of the Permanent Secretary, Ministry of Higher Education, Science, Research and Innovation (OPS MHESI), Thailand Science Research and Innovation (TSRI) and the National Research Council of Thailand (NRCT): NRCT5-RGJ63012-125.

## Conflict of interest

The authors declare that the research was conducted in the absence of any commercial or financial relationships that could be construed as a potential conflict of interest.

## Publisher’s note

All claims expressed in this article are solely those of the authors and do not necessarily represent those of their affiliated organizations, or those of the publisher, the editors and the reviewers. Any product that may be evaluated in this article, or claim that may be made by its manufacturer, is not guaranteed or endorsed by the publisher.
